# Automated identification of hip arthroplasty implants using artificial intelligence

**DOI:** 10.1038/s41598-022-16534-3

**Published:** 2022-07-16

**Authors:** Zibo Gong, Yonghui Fu, Ming He, Xinzhe Fu

**Affiliations:** 1 Department of Radiology, Shengjing Hospital of China Medical University, No. 36, Sanhao Street, Heping District, Shenyang City, 110004 People’s Republic of China; 2 Department of Orthopedics, Shengjing Hospital of China Medical University, No. 36, Sanhao Street, Heping District, Shenyang City, 110004 People’s Republic of China; 3 Lab for Information and Decision Systems, Massachusetts Institute of Technology, Boston, MA USA

**Keywords:** Radiography, Medical imaging, Health care

## Abstract

The purpose of this study was to develop and evaluate the performance of deep learning methods based on convolutional neural networks (CNN) to detect and identify specific hip arthroplasty models. In this study, we propose a novel deep learning-based approach to identify hip arthroplasty implants’ design using anterior–posterior images of both the stem and the cup. We harness the pre-trained ResNet50 CNN model and employ transfer learning methods to adapt the model for the implants identification task using a total of 714 radiographs of 4 different hip arthroplasty implant designs. Performance was compared with the operative notes and crosschecked with implant sheets. We also evaluate the difference in performance of models trained with the images of the stem, the cup or both. The training and validation data sets were comprised of 357 stem images and 357 cup radiographs across 313 patients and included 4 hip arthroplasty implants from 4 leading implant manufacturers. After 1000 training epochs the model classified 4 implant models with very high accuracy. Our results showed that jointly using stem images and cup images did not improve the classification accuracy of the CNN model. CNN can accurately distinguish between specific hip arthroplasty designs. This technology could offer a useful adjunct to the surgeon in preoperative identification of the prior implant. Using stem images or cup images to train the CNN can both achieve effective identification accuracy, with the accuracy of the stem images being higher. Using stem images and cup images together is not more effective than using images from only one perspective.

## Introduction

Total hip arthroplasty (THA) has been called the “Operation of the Century” because of its durability, reliability, and reproducibility in relieving pain and improving function in patients with coxarthrosis^1^. However, despite the clinical success of THAs, the number of revision THAs performed has increased with time. The etiology of the increase in the number of revision procedures is multifactorial. The increasing absolute number of primary arthroplasties, expansion of the indications to include younger and more active individuals, are all likely to contribute to overall revision rates. Projections based on population studies pointed to continued increases in the prevalence of revision procedures^2^. One of the critical steps in preoperative planning for revision of total hip arthroplasty is the identification of the failed implant. A recent survey of arthroplasty surgeons showed that surgeons spent approximately 20 min for each revision case to identify the implant preoperatively. 10% of implants could not be identified preoperatively with 2% not being identified intraoperatively. Failure to identify implants preoperatively resulted in additional requested implants, added surgical time, increased perioperative morbidity, and increased healthcare cost^3,4^.

Machine learning is an application of artificial intelligence (AI). It enables computers to find hidden insights without being explicitly programmed using algorithms that iteratively learn from the data^5,6^. In the field of general imaging and computer vision, deep learning is the leading machine learning tool^7^. Deep learning refers to techniques that build on developments in artificial neural networks in which multiple network layers are added to increase the levels of abstraction and performance^8^. Recently, deep learning, and in particular convolution neural networks (CNN), has shown groundbreaking results in a variety of general image recognition and computer-aided diagnosis tasks^9,10^. Within orthopedics, these powerful models already can reach human-level performance in the diagnosis of fractures^11,12^ and staging knee osteoarthritis (OA) severity^13^, which clearly indicates the possibility for using them in clinical practice in the near future.

We hypothesized that deep learning-based AI algorithms could facilitate the automated identification of hip arthroplasty implants, thereby aiding in preoperative planning and resulting in saved time and healthcare resources spent on this labor-intense task. Previous works have explored the potential of using CNN for hip arthroplasty implants classification where only images of the stem part of the implants were used as training data^14–16^. In this paper, we study CNN-based models for hip arthroplasty implants classification with images of both the stem part and the cup part of the implants. We evaluate the performance of the networks trained with the stem images and the cup images separately, and propose two ways to combine the stem images and the cup images. Our results show that CNN models based on stem images and cup images can both achieve satisfactory classification accuracy, while using images of the whole plants (with both stem and cup) is not more effective than using stem or cup images separately.

## Methods

### Study design and radiograph acquisition

After institutional review board approval, we retrospectively collected all radiographs taken between June 1, 2011 and Dec 1, 2020 at one university hospital. The images are collected by Neusoft PACS/RIS Version 5.5 on a personal computer running Windows 10. We confirm that all methods were performed in accordance with the relevant guidelines and regulations. Images were collected from surgeries performed by 3 fellowship-trained arthroplasty surgeons to ensure a variety of implant manufacturers and implant designs. At the time of collection, images had all identifying information removed and were thus de-identified. Implant type was identified through the primary surgery operative note and crosschecked with implant sheets. Implant designs were only included in our analysis if more than 30 images per model were identified^14^.

From the medical records of 313 patients, a total of 357 images were included in this analysis.

**Figure 1:**
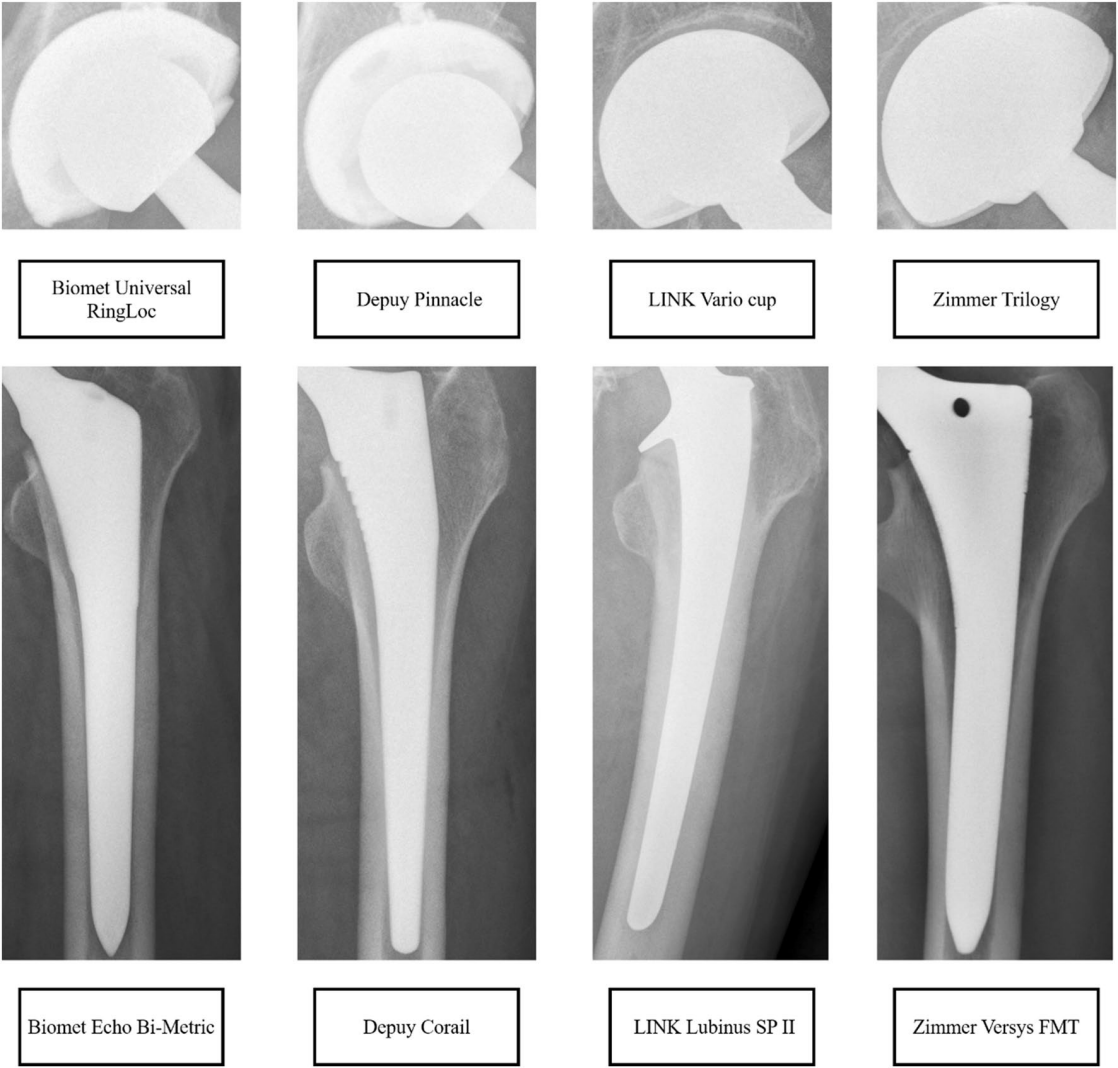
Demonstrated an example of cup and stem radiographs of each included implant design.

Although Zimmer and Biomet merged (Zimmer Biomet), these were treated as two distinct manufacturers. The following 4 designs from the four industry leading manufacturers were included: Biomet Echo Bi-Metric (Zimmer Biomet), Biomet Universal RingLoc (Zimmer Biomet), Depuy Corail (Depuy Synthes), Depuy Pinnacle (Depuy Synthes), LINK Lubinus SP II, LINK Vario cup, and Zimmer Versys FMT and Trilogy (Zimmer Biomet). Implant designs that did not meet the 30-implant threshold were not included. Figure 1 demonstrated an example of Cup and Stem anterior–posterior (AP) radiographs of each included implant design. The four types of implants are denoted as type A, type B, type C, and type D respectively in this paper.

### Overview of framework

We used convolutional neural network-based (CNN) algorithms for classification of hip implants. Our training data consist of images of anteroposterior (AP) view of the hips. For each image, we manually cut the image into two parts: the stem and the cup. We will train four CNN models, the first one using stem images (stem network), the second one using cup images (cup network), and the third one using the original uncut images (combined network). The fourth one is an integration of the models trained with stem network and the cup network (joint network).

Since the models involve millions of parameters, while our data set only contained less than one thousand images, it was infeasible to train a CNN model from scratch using our data. Therefore, we adopted the transfer learning framework to train our networks^17^. The transfer learning framework is a paradigm in the machine learning literature that is widely applied in scenarios where the training data is scarce compared to the scale of the model^18^. Under the transfer learning framework, the model is first initialized to some model pretrained with other data sets that contain enough data for a different but related task. Then, we tune the model using our data set by performing gradient descent (backward-propagation) only on the last two layers of the networks. As the parameters in the last two layers of the network are comparable with the size of our data set (for the target task), and the parameters in the previous layers have been tuned from the pre-trained model, the resulting network model can have satisfactory performance on the target task.

In our case, our CNN models we used are based on the established ResNet50 network pre-trained on the ImageNet data set^19^. The target task and our training data sets correspond to the images of the AP views of the hips (stem, cup, and combined).

**Figure 2:**
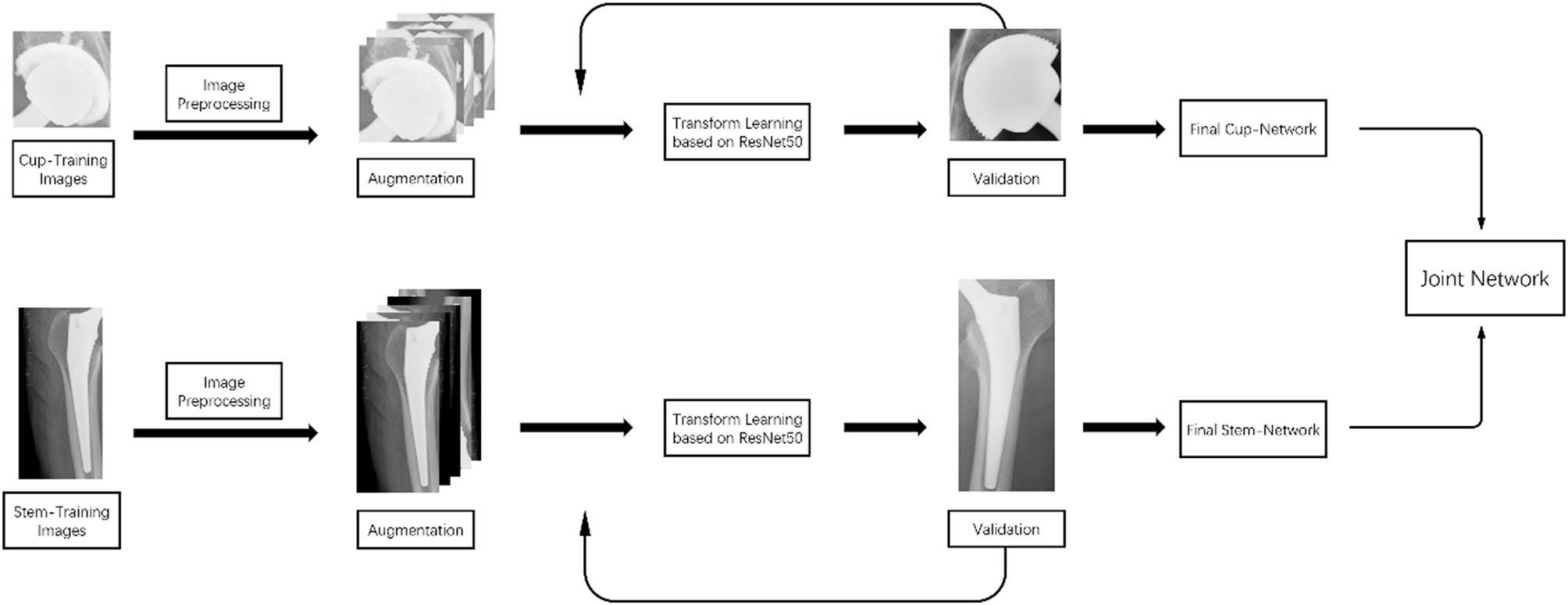
Overview of the framework of our deep learning-based method.

Figure 2 demonstrates the overview of the framework of our deep learning-based method.

### Dataset

Our dataset contained 714 images from 4 different kinds of implants.

### Image preprocessing

We followed standard procedures to pre-process our training data so that it could work with a network trained on ImageNet. We rescaled each image to a size of 224*224 and normalized it according to ImageNet standards. We also performed data augmentation, i.e., random rotation, horizontal flips, etc., to increase the amount of training data and make our algorithm robust to the orientation of the images.

### Dataset partition

We first divided the set of patients into three groups of sizes ~ 60% (group 1), ~ 30% (group 2), and ~ 10% (group 3). This split technique was used on a per-design basis to ensure the ratio of each implant remained constant. Next, we used the cup and stem images of patients in group 1 for training, those of patients in group 2 for validation, and those of patients in group 3 for testing. The validation set was used to compute cross-validation loss for hyper-parameter tuning and early stopping determination.

### Model training

We adopted the adaptive gradient method ADAM^20^ to train our models. Based on the cross-validation loss, we chose the hyper-parameters for ADAM as (learning rate $\mathrm{\alpha }$ = 0.001, ${\upbeta }_{1}=0.9, {\beta }_{2}=0.99, \epsilon ={10}^{-8},$ weight_decay = 0). The maximum number of epochs was 1000 and the batch size was 16. The early stopping threshold was set to 8. During the training process of each network, the early stopping threshold was hit after around 50 epochs. As we mentioned above, we trained four networks in total.

The first network is trained with the stem images, the second with the cup images. The third network is trained with the original uncut images, which is one way we propose to combine the power of stem images and cup images. We further integrate the first and the second network as an alternative way of jointly utilizing stem and cup images. The integration was done via the following logistic-regression based method. We collected the outputs of the stem network and the cup network (both are of the form of a 4-dimensional vector, with each element corresponding to the classification weight the network gives to the category of implants), and then fed them as the input to a two-layer feed-forward neural network, and trained the network with the data from the validation set. The integration is similar to a weighted-voting procedure among the outputs of the stem network and the cup network, with the weighting votes computed through the validation data set. Note that the above construction relied on our dataset division procedure, where the training set, validation set, and testing set, each contained the stem and cup images of the same set of patients. We referred to the resulting network constructed from the outputs of stem network and cup network as the “joint network”.

### Model testing

We tested our models (stem, cup, Joint) using the testing set. The prediction result on each testing image was a 4-dimensional vector, with each coordinate representing the classification confidence of the corresponding category of implants.

### Statistical analysis

Since we were studying a multi-class classification problem, we would directly present the confusion matrices of our methods on the testing data, and compute the operation characteristics generalized for multi-class classification.

### Ethical review committee

The institutional review board approved the study with a waiver of informed consent because all images were anonymized before the time of the study.

## Results

### Training progress

**Figure 3:**
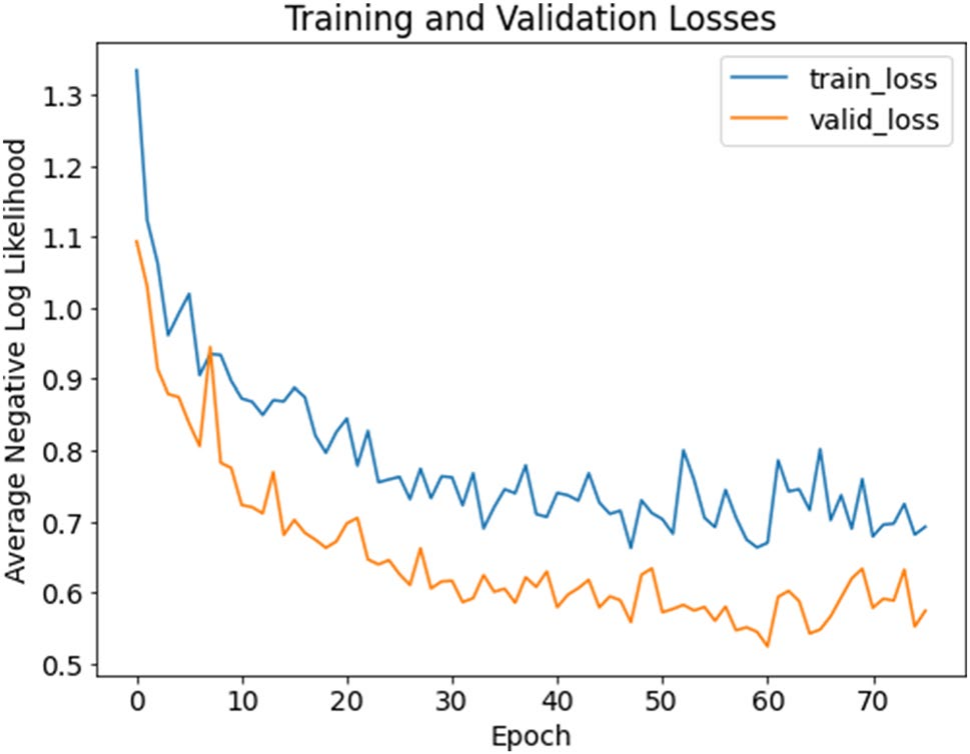
Training and validation losses curve of the cup-network.

The following figure (Fig. 3) shows the training progress of our method (we used the cup-network as an example). As the training proceeded, the network adjusted its parameters and learned the correct classification function as demonstrated by decrease in both training and validation loss.

### Testing performance

In this section, we evaluated our neural network models on the testing data sets. In the testing sets, we had in total 71 images. The classification results of the three neural network models (stem, cup, Joint) were presented in the form of tables. The column labels of each table indicated the ground-truth implant type, while row labels indicated the classification results. For example, the first row in the table of the stem-network indicated that the stem network classified 32 images with type-A plant as type A, and 3 images with type-A plant as type B.

### Stem-network

The classification results (Confusion Matrix) of the Stem-Network were shown in Table 1.

**Table 1:** Classification results (confusion matrix) of the stem-network.

Test results **Ground truths**	A	B	C	D
A	32	3	0	0
B	4	17	0	0
C	0	0	9	0
D	0	2	0	4

The stem network achieved an overall classification accuracy of 91.5%. Although the overall accuracy was high, the accuracy on some implants with scare training data was less satisfying. For example, its classification accuracy on implant type-B was about 69.6%.

### Stem-network

Table 1.

### Cup-network

The classification results (Confusion Matrix) of the Cup-Network are shown in Table 2.

**Table 2:** Classification results (confusion matrix) of the cup-network.

Test results **Ground truths**	A	B	C	D
A	28	6	1	0
B	6	15	0	0
C	0	0	9	0
D	0	3	0	3

The cup-network achieved a satisfying accuracy of 83.7%. However, its testing performance was dominated by that of the stem-network.

### Cup-network

Table 2.

### Combined network

The classification results (Confusion Matrix) of the Combined Network (trained with the original uncut images) are shown in Table 3.

**Table 3:** Classification results (confusion matrix) of the combined network.

Test results **Ground truths**	A	B	C	D
A	28	7	0	0
B	5	16	0	0
C	0	0	9	0
D	0	3	0	3

The combined network has an overall accuracy of 88.6%. However, from the experiments, we did not observe that training with the original uncut images achieve better performance than training with the stem images.

### Combined network

Table 3.

### Joint network

The classification results (Confusion Matrix) of the Joint Network are shown in Table 4.

**Table 4:** Classification results (confusion matrix) of the joint network.

Test results **Ground truths**	A	B	C	D
A	30	5	0	0
B	5	16	0	0
C	0	0	9	0
D	0	2	0	4

The join-network that essentially integrates the stem-network and the cup-network through a weighted-voting procedure and had an overall accuracy of 88.8%. Similar as for the combined network, from the experiments, we did not see that combining stem images with cup images had the potential to achieve the best of both worlds, and improved the classification performance of only using one category of image data. This can be mainly attributed to that the cup-network’s performance is dominated by the stem-network, which implies that the joint network cannot have better performance than the stem-network. However, the joint-network does have superior performance than the combined network, which suggests that using our procedure of integrating the outputs from the stem network and the cup network is a better way of utilizing both stem images and cup images than training a network with the uncut images that contain both the stem part and the cup part.

### Joint network

Table 4.

### Operation characteristics

For a multi-class classification method, let $M$ be its confusion matrix on the testing data. We used ${M}_{ij}$ to denote the entry on the $i$th row and $j$th column of $M$. Then, for each row (class), the recall and precision of the algorithm were defined as:

1 $$Precision_{i} = \frac{{M_{ii} }}{{\mathop \sum \nolimits_{j} M_{ji} }}$$

2 $$Recall_{i} = \frac{{M_{ii} }}{{\mathop \sum \nolimits_{j} M_{ij} }}$$

The precision (recall) of the algorithm was the average of its precision (recall) for each class.

Following the above definition, we had the following operating characteristics of the Stem-network, Cup-network, Combined-network and Joint-network (Table 5).

**Table 5:** Operation characteristics of our methods.

	Precision	Recall
Stem-network	0.915	0.847
Cup-network	0.837	0.754
Combined-network	0.886	0.765
Joint-network	0.888	0.821

Operating characteristics of the Stem-network, Cup-network, Combined-network and Joint-network.

## Discussion

Preoperative identification of arthroplasty implants prior to revision surgery is a difficult task and an essential step in preventing increases in perioperative morbidity and increased healthcare costs. The key finding of this study was that an artificial intelligence-based, deep learning CNN system could be trained to provide an automated identification of THA implants from radiographic images with near perfect accuracy.

To our knowledge, there are only 4 previous studies that have used deep learning for identifying hip arthroplasty implants from plain radiographs. One study by Kang et al.^21^ used a small sample sized dataset (170 AP hip radiographs) to distinguish between 29 hip implants. In particular, in their dataset, 24 hip implants had fewer than 10 samples and no implant has more than 20 samples.

Borjali et al.^15^ similarly applied a CNN to identify the design of nine different THA femoral implants from AP hip radiograph. Although limited by the volume of radiographs trained, this study showed that a deep-learning algorithm was capable of recognizing the unique femoral stem design features of a hip arthroplasty implant.

Karnuta et al.^14^ reported in the other study that their deep learning model using 1766 AP hip radiographs could discriminate successfully 18 hip implant models.

The study by Murphy et al.^16^ showed the impact artificial neural network architecture might play in test performance, ranging anywhere from 46.60 to 91.67% accuracy in test data depending on the architectures chosen.

However, all these four studies were only capable of identifying the femoral component of the implant, without paying attention to the identification ability of the deep-learning system to include the acetabular component of the implant.

In our study, the deep learning model developed from the AP view of hip radiographs represents the first advanced artificial intelligence model in identifying both the femoral component and the acetabular component of the implant from radiographs. Although the AP view of hip radiographs of different cup implants have similar appearances and are almost indistinguishable for human, the classification accuracy of our deep learning method is quite high (up to 83.7%). Furthermore, the accuracy of our method is higher than some of the previously proposed methods for stem implant identification. Finally, we propose two ways of combining the power of images of both the cup implants and stem implants. One is training with the original uncut images with both stem and cup parts, and the other one is integrating the outputs of stem networks and cup networks with a feed-forward network that mimics a weighted-voting procedure. Our results show that our deep-learning based models can achieve excellent classification results when trained with either images of stem implants, images of cup implants, or images of stem implants and cup implants together. Although in the results we observed that combining stem implants and cup implants did not lead to models with superior performance than only using the stem images, the joint-network we propose can be a meaningful procedure to utilize both stem implants and cup implants and may demonstrate superior performance in classification of implants of other models.

This study has several limitations.

First, the data come from only one institution, the number of images for each brand implant was highly varied because the contractual relationship between the single institution and different manufactures has been changing over the past 10 years. This could result in an imbalance in trained implant data.

Second, our algorithm was trained with only 4 hip arthroplasty implants; therefore, the data was limited to these models alone and should not be generalized to identify other models.

Third, clearly the CNN remains a less commonly studied model as it is technologically demanding and requires access to numerous data, a team involving a surgeon, radiologist, and computer scientist to apply the deep learning models prior to implementing this type of solution in clinical practice.

## Conclusion

In conclusion, the technology behind machine learning is exponentially advancing and the enthusiasm for AI in healthcare is growing. With this study, we have demonstrated that a deep learning algorithm can identify the design of 4 different hip arthroplasty implants from AP hip radiograph. These findings suggest that this technology has the potential to classify hip implants prior to revision surgery, thus saving significant time, and reducing perioperative morbidity and healthcare cost. It is hoped that it can be used to collect large-scale implant information and may be applied to mobile phone applications in the future^22^.

## Data Availability

The datasets generated during and analyzed during the current study are not publicly available due to the requirement of The Ethics Committee of Shengjing Hospital but are available from the corresponding author on reasonable request.

## Abbreviations

AI: Artificial intelligence
CNN: Convolutional neural networks
THA: Total hip arthroplasty
OA: Osteoarthritis
AP: Anterior-posterior
